# Comparison of cancer incidence among patients with rheumatic disease: a retrospective cohort study

**DOI:** 10.1186/s13075-014-0428-x

**Published:** 2014-08-28

**Authors:** Sung Hae Chang, Jin Kyun Park, Yun Jong Lee, Ji Ae Yang, Eun Young Lee, Yeong Wook Song, Eun Bong Lee

**Affiliations:** Division of Rheumatology, Department of Internal Medicine, Seoul National University College of Medicine, 101, Daehak-ro, Jongno-gu, Seoul 110-744 Korea

## Abstract

**Introduction:**

Rheumatic diseases (RDs) are associated with different cancers; however, it is unclear whether particular cancers are more prevalent in certain RDs. In the present study, we examined the relative incidence of several cancers in a single homogeneous cohort of patients with different RDs.

**Methods:**

Patients (*N* = 3,586) diagnosed with rheumatoid arthritis (RA), systemic lupus erythematosus (SLE), systemic sclerosis (SSc), dermatomyositis (DM) or polymyositis were included. Cancer diagnosis was based on histopathology. The 2008 Korean National Cancer Registry served as the reference for calculating standardized incidence ratios (SIRs).

**Results:**

During the follow-up period of 31,064 person-years, 187 patients developed cancer. RA and SLE patients showed an increased risk of non-Hodgkin’s lymphoma (SIR for RA patients = 3.387, 95% confidence interval (CI) = 1.462 to 6.673; SIR for SLE patients = 7.408, 95% CI = 2.405 to 17.287). SLE patients also had a higher risk of cervical cancer (SIR = 4.282, 95% CI = 1.722 to 8.824). SSc patients showed a higher risk of lung cancer (SIR = 4.917, 95% CI = 1.977 to 10.131). Endometrial cancer was increased only in patients with DM (SIR = 30.529, 95% CI = 3.697 to 110.283). RA patients had a lower risk for gastric cancer (SIR = 0.663, 95% CI = 0.327 to 0.998). The mean time between the RD and cancer diagnoses ranged from 0.1 to 16.6 years, with the shortest time observed in patients with DM (2.0 ± 2.1 years).

**Conclusions:**

Different RDs are associated with particular cancers. Thus, cancer surveillance tailored to specific RDs might be beneficial.

**Electronic supplementary material:**

The online version of this article (doi:10.1186/s13075-014-0428-x) contains supplementary material, which is available to authorized users.

## Introduction

Rheumatic diseases (RDs) result from immune dysfunction, which leads to chronic systemic inflammation and damage to multiple organs. Precancerous cells can escape cancer surveillance associated with impaired immune response [1]. Chronic inflammation, along with the associated production of cytokines and growth factors, may promote the progression of precancerous cells to a clinically relevant cancer [2]. Indeed, there is a robust link between cancer and RDs. Patients with rheumatoid arthritis (RA), systemic lupus erythematosus (SLE) and Sjögren’s syndrome (SS) have a higher risk of developing lymphoma [3-5], and those with systemic sclerosis (SSc) tend to have a higher risk of developing lung cancer [6,7]. These studies suggest that the type of RD a patient has is associated with an increased risk of developing certain cancers. This may be due to the nature of the underlying immune dysfunction and the extent of involved organ damage.

However, previous studies have shown marked variations in the estimated incidence of cancer in patients with the same type of RD. This is likely due to differences in genetic background, environmental factors and available treatment options. For example, patients with dermatomyositis (DM) in Scandinavia show an increased risk of lung or endometrial cancer [8], whereas those in Taiwan show an increased risk of head and neck cancer [9]. The cancer risk even differs between different Asian populations with the same RD. Taiwanese RA patients have an increased risk of kidney and vaginal cancer, but no such associations are observed in Japanese RA patients [10,11]. In addition to the heterogeneity of the study populations, the methodological differences in case ascertainment likely contribute to the varying SIR estimates across studies. Population-based studies using National Health Insurance claims or hospital discharge registry data might reveal rare cancers at the expense of diagnostic accuracy of RDs and cancer, especially when longitudinal follow-up data are not available. Thus, comparison of cancer incidences from multiple studies would not conclusively address the question whether a particular RD is associated with a higher risk of developing a certain cancer. Our aim in the present study was to compare the relative incidence of several common cancers in different RDs in a single homogeneous cohort.

## Methods

### Patients

As patients with a rare or critical illness, including RDs and cancer, are entitled to reimbursement of a majority of their medical copayments, diagnosis of RDs and cancer is subjected to comprehensive clinical and laboratory assessments in Korea. Thus, when the same International Classification of Diseases (ICD) codes are entered repeatedly over a certain period of time for billing and reimbursement purposes, diagnosis of RD is accurate with a high probability.

We first selected patients with the same ICD-10 code for a specific RD (that is, RA (M 05.X, M 06.X), SLE (M 32.X), SSc (M 34.X), DM (M 33.0, 33.1, 33.9), PM (M 33.2)] for more than 6 months during their longitudinal medical care at Seoul National University Hospital (SNUH) between January 2000 and April 2012. Next, the medical records of all selected patients with a diagnosis of probable RD were reviewed. Only the patients with a definitive RD diagnosis who fulfilled the criteria for RA, SLE, SSc, DM or PM were enrolled in this study (Additional file 1: Figure S1) [12-15]. Patients who developed cancer before or within 1 month of RD diagnosis were excluded.

The 2008 Korean National Cancer Registry, provided by the Ministry of Health and Welfare of Korea [16], was used as the reference for calculating the standardized incidence ratios (SIRs). The study was approved by the Institutional Review Board (IRB) of SNUH. Obtaining a patient consent was waived by the IRB, as this study involved no more than minimal risk as a retrospective study and no identifiable information was used.

### Cancer diagnosis

Histopathology reports were available in all cancer cases. A complete data set regarding cancer histopathology was obtained from the medical records.

### Statistical analysis

The expected number of cancers was calculated by multiplying the number of patients in the study cohort by the age- and sex-matched cancer incidence rate for Koreans obtained from the 2008 Korean National Cancer Registry. When a patient was diagnosed with two or more cancers during follow-up, each was considered as an individual event. The SIR was calculated as the ratio of the observed to the expected number of cancers, and the 95% confidence interval (95% CI) was calculated based on the assumption that the observed findings followed a Poisson distribution [17]. Analysis of variance or Student’s *t*-tests were used for group comparisons as appropriate. The results were expressed as the mean ± standard deviation. A *P*-value ≤0.05 was considered statistically significant. All analyses were performed using SPSS version 16.0 statistical software (IBM SPSS, Chicago, IL, USA).

## Results

### Study population

A total of 3,586 patients (84.0% female) with a definitive RD diagnosis were identified and enrolled in the study. The mean duration of follow-up was 7.8 ± 4.5 years, with a total follow-up of 31,064 person-years (PYs). All patients were ethnic Koreans. The mean age at the time of RD diagnosis was 45.9 ± 15.2 years. RA was the most common diagnosis (2,104 patients; 58.7%), followed by SLE (1,052; 29.3%), SSc (274; 7.6%), DM (107; 3.0%) and PM (49; 1.4%). Of these, 185 patients were diagnosed with a malignancy, and 2 patients had 2 cancers, with an overall cancer incidence of 0.60/100 PYs. The mean time between the RD diagnosis and that of cancer ranged from 0.1 to 16.6 years, with the shortest time observed in patients with DM (2.0 ± 2.1 years). Notably, 5 (50.5%) of the 10 malignancies were diagnosed within 1 year of the DM diagnosis (Table 1).

**Table 1:** **Demographic characteristics of the rheumatic disease patients** ^**a**^

	**RA (** ***n*** **= 2,104)**	**SLE (** ***n*** **= 1,052)**	**SSc (** ***n*** **= 274)**	**DM (** ***n*** **= 107)**	**PM (** ***n*** **= 49)**
Age at RD diagnosis, yr, mean (SD)	51.0 (13.3)	35.0 (13.4)	48.5 (14.0)	45.5 (13.4)	47.9 (13.7)
Sex, female (%)	1,716 (81.6)	935 (88.9)	240 (87.6)	81 (75.7)	40 (81.6)
Mean follow-up duration, yr (SD)	7.4 (4.2)	8.9 (4.8)	6.6 (4.5)	6.1 (4.8)	6.7 (4.8)
Total follow-up duration in person-years	17,436	10,410	2,088	758	372
Cancer, *n* (%)	106 (5.0)	53 (5.0)	16 (5.8)	10 (9.3)	2 (4.1)
Age at cancer diagnosis, yr, mean (SD)	62.8 (11.7)	47.3 (14.7)	55.5 (11.9)	58.1 (14.6)	65.8 (0.3)
Time between RD and cancer, yr, mean (SD)	5.0 (3.5)	7.4 (4.0)	5.8 (3.5)	2.0 (2.1)	5.0 (6.1)

^a^DM, Dermatomyositis; PM, Polymyositis; RA, Rheumatoid arthritis; RD, Rheumatic disease; SD, Standard deviation; SLE, Systemic lupus erythematosus; SSc, Systemic sclerosis.

### Cancer incidence

**Figure 1:**
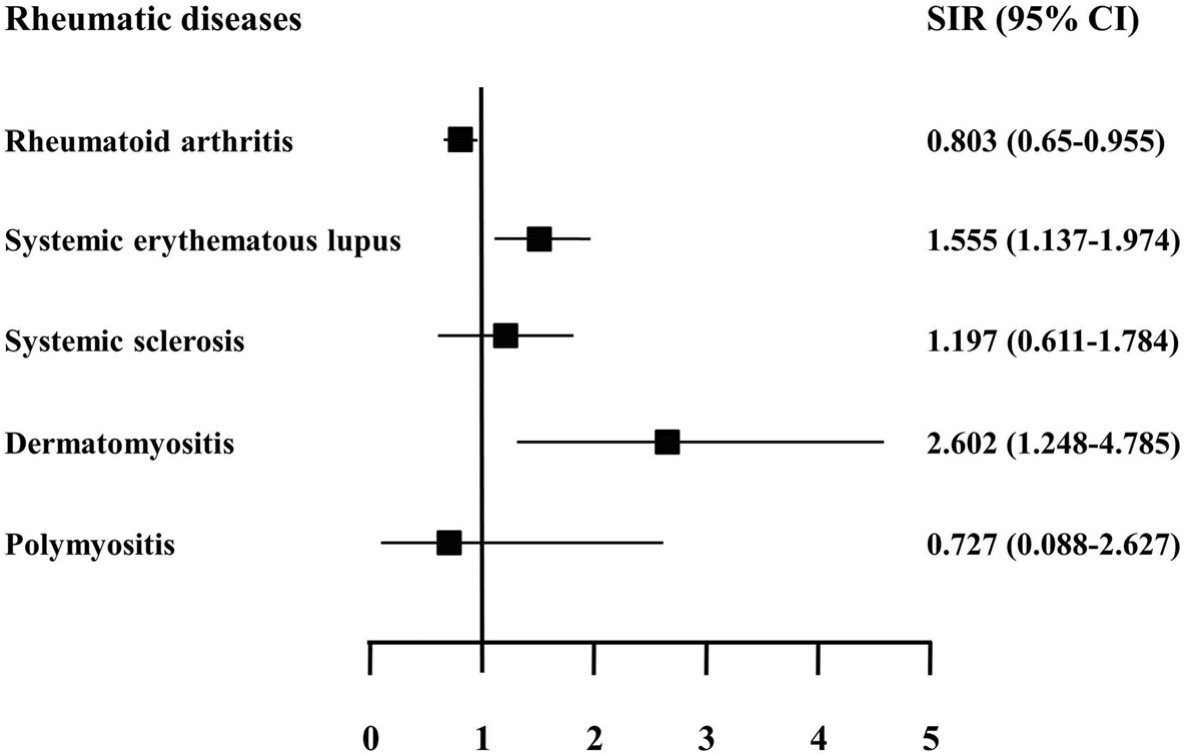
**Cancer incidence in patients with rheumatic disease.** The incidence of cancer was higher in patients with systemic lupus erythematosus and dermatomyositis, but lower in those with rheumatoid arthritis, compared with that in the general population. SIR, Standardized incidence ratio; CI, Confidence interval.

The incidence of cancer varied between patients with different types of RD. Compared with the incidence in the general population, the cancer incidence was higher in patients with SLE (SIR = 1.555; 95% CI, 1.137 to 1.974) and DM (SIR = 2.602; 95% CI, 1.248 to 4.785), but lower in patients with RA (SIR = 0.803; 95% CI, 0.65 to 0.955) (Figure 1).

### Association between individual rheumatic diseases and particular cancers

The SIR for NHL was increased in patients with RA (SIR = 3.387; 95% CI, 1.462 to 6.673) and in patients with SLE (SIR = 7.408; 95% CI, 2.405 to 17.287). The incidence of cervical cancer was increased only in patients with SLE (SIR = 4.282; 95% CI, 1.722 to 8.824), whereas that of lung cancer was increased only in patients with SSc (SIR = 4.282; 95% CI, 1.722 to 8.824). A higher incidence of endometrial cancer was estimated only in patients with DM (SIR = 30.529; 95% CI, 3.697 to 110.283). The incidence of gastric cancer was estimated to be lower in patients with RA (SIR = 0.663; 95% CI, 0.327 to 0.997) (Table 2).

**Table 2:** **Standardized incidence ratios for each cancer according to type of rheumatic disease** ^**a**^

**Site of cancer**		**RA**	**SLE**	**SSc**	**DM**	**PM**
Breast	O/E	10/12.509	4/5.883	0/1.653	0/0.492	0/0.282
	SIR (CI)	0.799 (0.304 to 1.295)	0.68 (0.185 to 1.741)	0 (0 to 2.231)	0 (0 to 7.492)	0 (0 to 13.088)
Cervical	O/E	4/3.786	**7/1.635**	0/0.47	0/0.136	0/0.085
	SIR (CI)	1.056 (0.288 to 2.705)	**4.282 (1.722 to 8.824)**	0 (0 to 7.846)	0 (0 to 27.198)	0 (0 to 43.353)
Colorectal	O/E	13/18.514	5/3.933	1/1.803	0/0.491	0/0.386
	SIR (CI)	0.702 (0.32 to 1.084)	1.271 (0.413 to 2.967)	0.555 (0.014 to 3.09)	0 (0 to 7.518)	0 (0 to 9.55)
Endometrial	O/E	0/1.883	0/0.742	0/0.238	**2/0.066**	0/0.044
	SIR (CI)	0 (0 to 1.959)	0 (0 to 4.974)	0 (0 to 15.512)	**30.529 (3.697 to 110.283)**	0 (0 to 84.476)
Lung	O/E	14/15.667	0/2.637	**7/1.424**	2/0.363	0/0.319
	SIR (CI)	0.894 (0.489 to 1.499)	0 (0 to 1.399)	**4.917 (1.977 to 10.131)**	5.514 (0.668 to 19.917)	0 (0 to 11.546)
NHL	O/E	**8/2.462**	**5/0.675**	1/0.242	1/0.071	0/0.049
	SIR (CI)	**3.387 (1.462 to 6.673)**	**7.408 (2.405 to 17.287)**	4.141 (0.105 to 23.071)	14.088 (0.357 to 78.491)	0 (0 to 74.764)
Stomach	O/E	**15/22.625**	3/5.027	2/2.236	1/0.614	1/0.473
	SIR (CI)	**0.663 (0.327 to 0.998)**	0.597 (0.123 to 1.744)	0.898 (0.109 to 3.245)	1.629 (0.041 to 9.076)	2.113 (0.054 to 11.774)
Thyroid	O/E	14/14.957	11/7.111	0/1.829	1/0.633	0/0.318
	SIR (CI)	0.936 (0.446 to 1.426)	1.547 (0.633 to 2.461)	0 (0 to 2.017)	1.58 (0.04 to 8.805)	0 (0 to 11.601)

^a^DM, dermatomyositis; E, expected cases; NHL, Non-Hodgkin’s lymphoma; O, observed cases; PM, polymyositis; RA, rheumatoid arthritis; SLE, systemic lupus erythematosus; SSc, systemic sclerosis. Numbers in parentheses represent the 95% confidence interval (CI). CIs in bold do not include 1. Data in bold are statistically significant.

## Discussion

Impaired immune surveillance in patients with autoimmune disease might allow precancerous cells to grow, thereby increasing the overall risk of developing a clinically relevant cancer [1]. Indeed, an association between particular cancers and specific RDs has been reported, and the risk of developing a specific cancer appears to vary markedly depending on the RD [18,19]. However, as previous studies have shown marked variations in the estimated incidence of the same cancer in patients with the same RD, comparison of estimated SIRs from different studies has limitations. To the best of our knowledge, the present study is the first in which the incidence of common cancers in a single homogeneous cohort of patients with different RDs has been estimated and compared.

In line with previous reports, our data show that patients with RA and SLE, but not those with SSc, DM or PM, had an increased risk of developing lymphoma, suggesting that the strong activation of lymphoid tissue is a driving force (similar to the finding that chronic viral or bacterial infections increase the risk of developing lymphoma) [20]. Indeed, overexpression of B lymphocyte simulator (BLyS) is associated with lymphoproliferative disorders in patients with SS [21]. Patients with RA and SLE exhibit high levels of BLyS [22,23].

Consistent with prior studies, lung cancer risk was higher in patients with SSc [24,25]. Reduced forced vital capacity and pulmonary fibrosis are associated with an increased risk of developing lung cancer, suggesting that the extent of lung damage may predict subsequent cancer development [7]. In the present study, the incidence of lung cancer in patients with DM, which often involves severe interstitial lung disease, appeared to be increased, although the increase did not reach statistical significance.

The overall risk of developing cancer was lower for RA patients, in part due to the lower incidence of stomach cancer (Table 2). This is striking because stomach cancer is common in Korea, with an incidence of 43.8 per 100,000, which is due, at least in part, to the high prevalence of *Helicobacter pylori* infection [26]. The lower overall incidence of gastric cancer in RA patients could be explained by the increased use of nonsteroidal anti-inflammatory drugs (NSAIDs), which reduce proliferative stimuli and may slow down the progression of a precancerous lesion [27]. Wu *et al*. reported that regular NSAID use for 6 months or longer was associated with a lower risk of developing a gastric cancer [28]. In a pilot study, we compared NSAID use between RA patients with gastric cancer and those without cancer. Thirteen of fifteen RA patients with gastric cancer and forty of forty-five age- and sex-matched RA patients without gastric cancer took NSAIDs on regular basis for 6 months or longer (86.7% vs. 88.9%, *P* = 1.0 by Fisher’s exact test). Although we could not confirm the protective effect of NSAIDs on gastric cancer in our cohort, it is still possible that gastrointestinal complaints associated with frequent use of NSAIDs might lead to early diagnosis and eradication of subclinical *H. pylori* infections in RA patients, thereby reducing the overall incidence of gastric cancer.

A population-based study using data from Sweden, Denmark and Finland showed that ovarian and cervical cancers were particularly increased in DM and PM [8]. In our present study, such associations were not found. Instead, a higher SIR for endometrial cancer was observed in DM patients.

Strikingly, five of the ten DM patients developed cancer within 1 year of their DM diagnosis (Additional file 1: Table S1), with a mean time between RD and cancer diagnosis of 2.0 ± 2.1 years as compared to 7.4 ± 4.2 years, 8.9 ± 4.8 years and 6.6 ± 4.5 years for RA, SLE and SSc patients, respectively. The tight temporal relationship between DM and cancer suggests that a DM subset might be triggered as an immunologic response to cancer, similar to what is seen in SSc patients with autoantibodies against RNA polymerase III. In those SSc patients, cancer was detected before or simultaneously with their SSc diagnosis, and their peripheral T cells reacted to the peptides derived from mutated RNA polymerase III [29]. It is tempting to speculate that preclinical malignancies may initiate and drive the development of DM through molecular mimicry between mutated cancer protein and self-antigen, because treatment of the underlying cancer has been reported to improve DM [30].

There was no association between thyroid cancer and RD or between breast cancer and RD, and their SIRs were similar across RD as well (Table 2). It appears that having RD does not increase the risk of developing cancers that are common in the general population, particularly if the cancer site is not an organ affected by the systemic inflammation associated with RD.

A strength of the present study as a single center study is the homogeneity of study population and the high quality of the clinical data obtained in conjunction with a relatively long follow-up of 31,064 PYs. This study design allowed us to better estimate the “pure” effect of individual RDs on cancer development. However, our study has several limitations. Compared to a population-based study, the cohort size was relatively small, with a wide CI for SIR estimates and a low sensitivity for detecting rare cancers. Because our hospital is a major tertiary referral center, the patients in our cohort may have had more severe disease and multiple comorbidities; therefore, they may have been monitored more closely and treated more quickly or differently, thereby leading to possible treatment bias. As this was a retrospective study, another limitation is that information on the cancer in some patients with RD could not be captured completely, especially when patients received their cancer treatment outside our center, possibly contributing to the lower SIR of cancers in patients with RD. Last but not least, we did not examine the treatment effects on cancer development. For example, pulmonary fibrosis may be a side effect of methotrexate and can predispose a patient to developing lung cancer. Cyclophosphamide can induce the development of secondary cancers [31]. In addition, the prolonged use of corticosteroids and immunosuppressants can increase infection rates, which may then increase cancer risk [32].

## Conclusions

Different RDs appear to be associated with particular cancers. Therefore, RD patients might benefit from cancer surveillance that is tailored to their RD.

## Additional file

Additional file 1: Figure S1. Study population. **Table S1.** Characteristics of dermatomyositis patients with cancer.

## Abbreviations

ACR: American College of Rheumatology
BLyS: B lymphocyte simulator
CI: Confidence interval
DM: Dermatomyositis
IRB: Institutional Review Board
NHL: Non-Hodgkin’s lymphoma
PM: Polymyositis
PY: Person-year
RA: Rheumatoid arthritis
RD: Rheumatic disease
SD: Standard deviation
SIR: Standardized incidence ratio
SLE: Systemic lupus erythematosus
SSc: Systemic sclerosis
