# Supplementary material for: Comparison of cancer incidence among patients with rheumatic disease: a retrospective cohort study
Source: Arthritis Res Ther. 2014 Aug 28;16(4):428. doi: 10.1186/s13075-014-0428-x (PMC4295295; doi:10.1186/s13075-014-0428-x)
Supplement: Additional file 1: Figure S1. — Study population. Table S1. Characteristics of dermatomyositis patients with cancer. [file 13075_2014_428_MOESM1_ESM.doc]

**Figure S1.** Study population


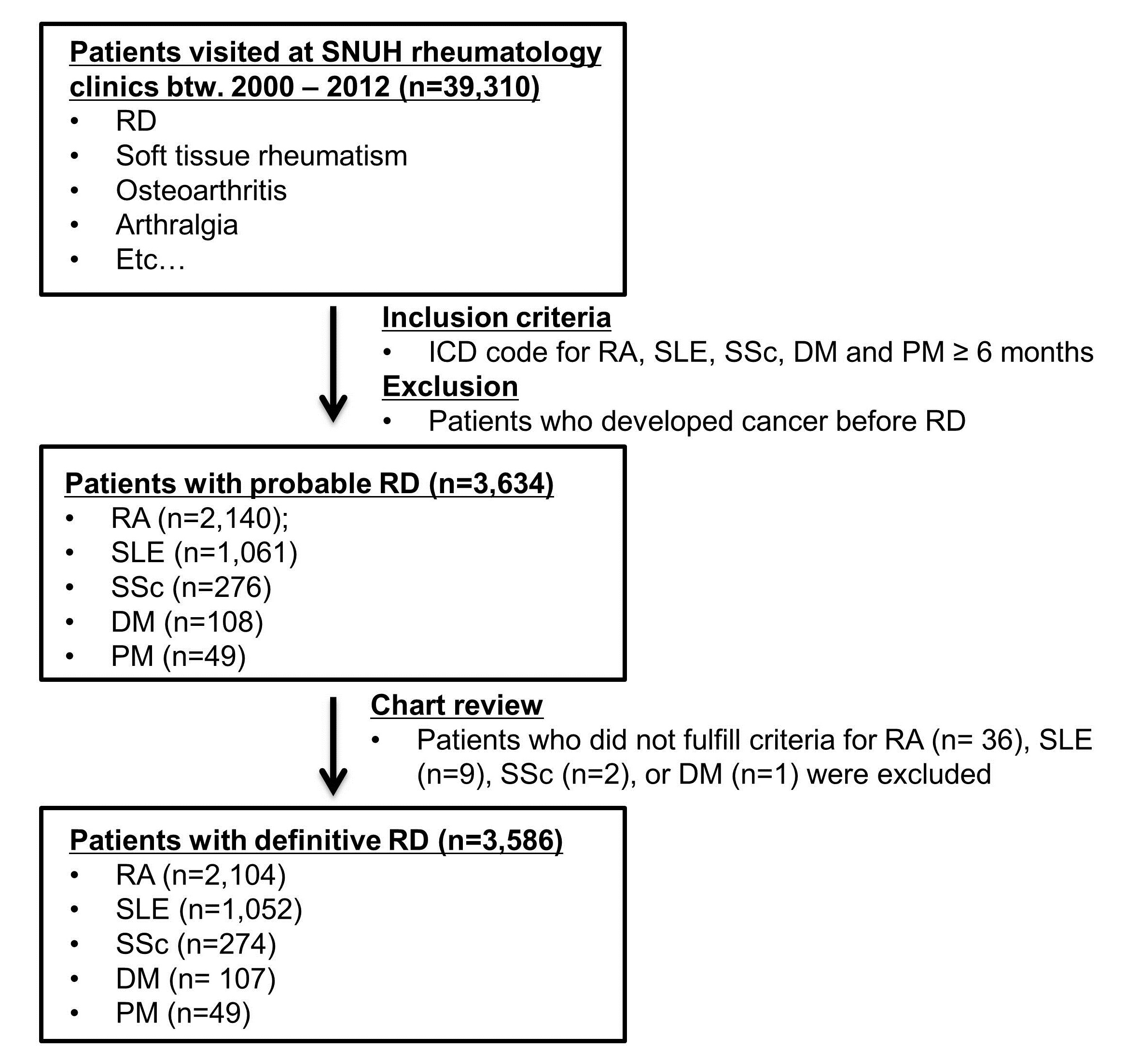


**Table S1. Dermatomyositis patients with cancer.**

|  | Sex | Site of cancer | Age at RD Dx (yrs) | Age at cancer Dx (yrs) | Time between RD and cancer Dx (yrs) |
| --- | --- | --- | --- | --- | --- |
| Patient 1 | F | MUO | 73.6 | 75 | 1.6 |
| Patient 2 | F | Bladder | 67.7 | 72 | 4.2 |
| Patient 3 | F | Endometrium | 58.3 | 59 | 0.8 |
| Patient 4 | M | Lung | 52.6 | 60 | 7.0 |
| Patient 5 | M | NHL | 36.9 | 37 | 0.6 |
| Patient 6 | M | Stomach | 65.4 | 67 | 2.0 |
| Patient 7 | F | Thyroid | 29.2 | 31 | 2.0 |
| Patient 8 | M | Lung | 68.4 | 69 | 0.3 |
| Patient 9 | F | Endometrium | 60.2 | 61 | 1.0 |
| Patient 10 | F | Ovary | 49.3 | 49 | 0.1 |

F, female; M, male; RD, rheumatic disease; Dx, diagnosis; yrs, years; MUO, malignancy of unknown origin; NHL, non-Hodgkin’s lymphoma.
